# Expansion of an Australian food composition database to estimate plant and animal intakes

**DOI:** 10.1017/S0007114523001101

**Published:** 2023-12-14

**Authors:** Jordan Stanford, Sarah McMahon, Kelly Lambert, Karen E. Charlton, Anita Stefoska-Needham

**Affiliations:** 1 School of Medical, Indigenous and Health Sciences, University of Wollongong, Northfields Ave, Wollongong, New South Wales, 2522, Australia; 2 Illawarra Health and Medical Research Institute, Wollongong, NSW 2522, Australia

**Keywords:** Plant-based diets, Food analysis, Food composition database, AUSNUT 2011–2013, Diet Quality Index

## Abstract

Despite evidence for favourable health outcomes associated with plant-based diets, a database containing the plant and animal content of all foods eaten is required to undertake a reliable assessment of plant-based diets within a population. This study aimed to expand an existing Australian food database to include the plant and animal content of all whole foods, beverages, multi-ingredient products and mixed dishes. Twenty-three plant- and animal-based food group classifications were first defined. The food servings per 100 g of each product were then systematically calculated using either a recipe-based approach, a food label-based approach, estimates based on similar products or online recipes. Overall, 4687 (83·5 %) foods and beverages were identified as plant or plant-containing products, and 3701 (65·9 %) were animal or animal-containing products. Results highlighted the versatility of plant and animal ingredients as they were found in various foods across many food categories, including savoury and sweet foods, as well as discretionary and core foods. For example, over 97 % of animal fat-containing foods were found in major food groups outside the AUSNUT 2011–2013 ‘fats and oils’ group. Surprisingly, fruits, nuts and seeds were present in a greater percentage of discretionary products than in core foods and beverages. This article describes a systematic approach that is suitable for the development of other novel food databases. This database allows more accurate quantitative estimates of plant and animal intakes, which is significant for future epidemiological and clinical research aiming to investigate plant-based diets and their related health outcomes.

Plant-based diets are gaining popularity as a growing body of evidence indicates positive impacts on both human and planetary health^(1,2)^. Prior research investigating the health effects of plant-based diets has assumed that all plant-based diets are equally healthy^(3,4)^. This is partly owing to a lack of overall diet quality assessments, resulting in less healthy foods such as refined grains and sweets being inadvertently included as plant foods. Plant-based diet indices have recently been developed^(5,6)^ to distinguish between healthy and less healthy plant foods, though the majority are designed for FFQ and are incompatible with the data output from all other dietary collection methods, including 24-hour recalls, food records and diet histories. Additionally, current methods only analyse foods derived entirely from plants or products primarily made from plants, disregarding the various ingredients found in multi-ingredient foods and mixed dishes.

A database containing the plant and animal content of all foods eaten is required to undertake a reliable assessment of plant-based diets within a population. The Australian Dietary Guidelines (ADG) database^(7)^ (developed by Food Standards Australia New Zealand (FSANZ), with input from the Australian Bureau of Statistics (ABS)), is currently the primary source of data used to estimate population intakes of certain plant and animal food groups, such as whole grains, vegetables and red meat. The ADG database was designed to assess the degree of adherence to the 2013 Australian Dietary Guidelines recommendations, using food consumption data from the 2011–2012 National Nutrition and Physical Activity Survey (NNPAS)^(8)^, a sub-component of the Australian Health Survey. Based on a nationally representative survey of 12 153 people, the NNPAS provides the most recent dietary intake data for Australians^(8)^.

The analysis of reported plant and animal intakes is limited in the current ADG database, with several NNPAS foods and drink items not even classified because they fall outside of the five food groups used for dietary guidance in Australia. Examples of such exclusions are widely consumed plant and animal foods that are considered ‘discretionary foods and beverages’ as per the ADG definitions^(9)^. This refers to products that are usually rich in saturated fat, sugar and salt, and do not fit within the ADG’s five core food groups. Although these products are not necessary for meeting nutrient requirements, they may enhance the overall pleasure of eating if consumed occasionally or in small amounts. Examples of such products are butter, creams, coconut oils, gravies and maple syrups. Core foods, on the other hand, are nutrient-rich foods that are considered necessary for health (e.g., fruit, vegetables, grains, lean meat and dairy). In addition, some plant or animal ingredients found in a range of multi-ingredient products and mixed dishes are overlooked, such as the egg content in the item: *Pasta, white wheat flour, plain, homemade, boiled, no added salt.*

Given the lack of a comprehensive method to accurately classify foods according to whether they are plant or animal-based, this study aimed to expand an existing database to better distinguish and calculate plant and animal intakes from whole foods, multi-ingredient products and mixed dishes. As a result of this updated database, other dietary data inputs (such as data from diet histories and 24-hour recalls) can now be used to assess diet quality using existing plant-based diet quality indices. The development of such a database also offers the potential to readily calculate plant or animal intakes down to the ingredient level, which is of significance for future epidemiological and clinical research aiming to investigate plant-based diets and their related health outcomes. In particular, this research could be important for developing biomarkers of plant-based dietary patterns and expanding studies investigating ultra-processed foods within the context of plant-based diets.

## Materials and methods

To develop this database, the ADG database^(7)^ was expanded to identify the plant and animal content of whole foods, multi-ingredient products and mixed dishes. The ADG database was built upon the food composition survey database AUSNUT 2011–2013^(10)^, which includes all 5740 foods and beverages reported within the 2011–2012 NNPAS. The ADG database presents values for the number of respective servings per food group in 100 g of each classified food or beverage product. This allows users to determine the number of servings for each food group based on the specific amount consumed. For example, if an individual consumes 20 g of hollandaise sauce, they can divide the servings per 100 g in the database by 100 to obtain the servings per 1 g. The resulting value can then be multiplied by 20 to calculate the number of servings for each food group. The same process can be applied for an accurate quantification if someone consumes 50 g. However, as mentioned in the introduction, several NNPAS food and beverage items in the ADG, as well as multi-ingredient and mixed dishes, remain unclassified and/or do not include complete plant and animal content information.

In addition to the ADG database^(7)^, three other supporting resources were also used for the database development, including the AUSNUT 2011–2013 Food Details database file^(11)^, the AUSNUT 2011–2013 Food Recipe file^(12)^ and the AUSNUT 2011–2013 Food Nutrient Database file^(10)^. All four resources contained the food ID, survey ID and food name, which were required to match relevant information amongst the different files for all 5740 foods and beverages. The AUSNUT 2011–2013 Food Details database file^(11)^ provided additional information regarding the description of the food, inclusion criteria that detail specific commercial products that are representative of each food identification code, and sampling details for each product. The AUSNUT 2011–2013 Food Recipe file^(12)^ provides the ingredient ID, ingredient name and ingredient weight (g) required to calculate the portion of ingredients in multi-ingredient products and mixed dishes. Finally, the AUSNUT 2011–2013 Food Nutrient Database file^(10)^ provides relevant nutrient content for all 5740 foods and beverages, including energy (kJ), sodium (Na; mg) and added sugars (g).

The development of this database consisted of two key phases. Phase one was to define plant and animal food group classifications and establish specific serving sizes for each of the classifications relevant to existing plant-based diet indices. The second phase involved identifying and classifying each of the AUSNUT 2011–2013 food and beverages against the food group classifications defined in phase one to calculate the plant and animal content of each item.

### Defining plant and animal food group classifications

**Fig. 1.**
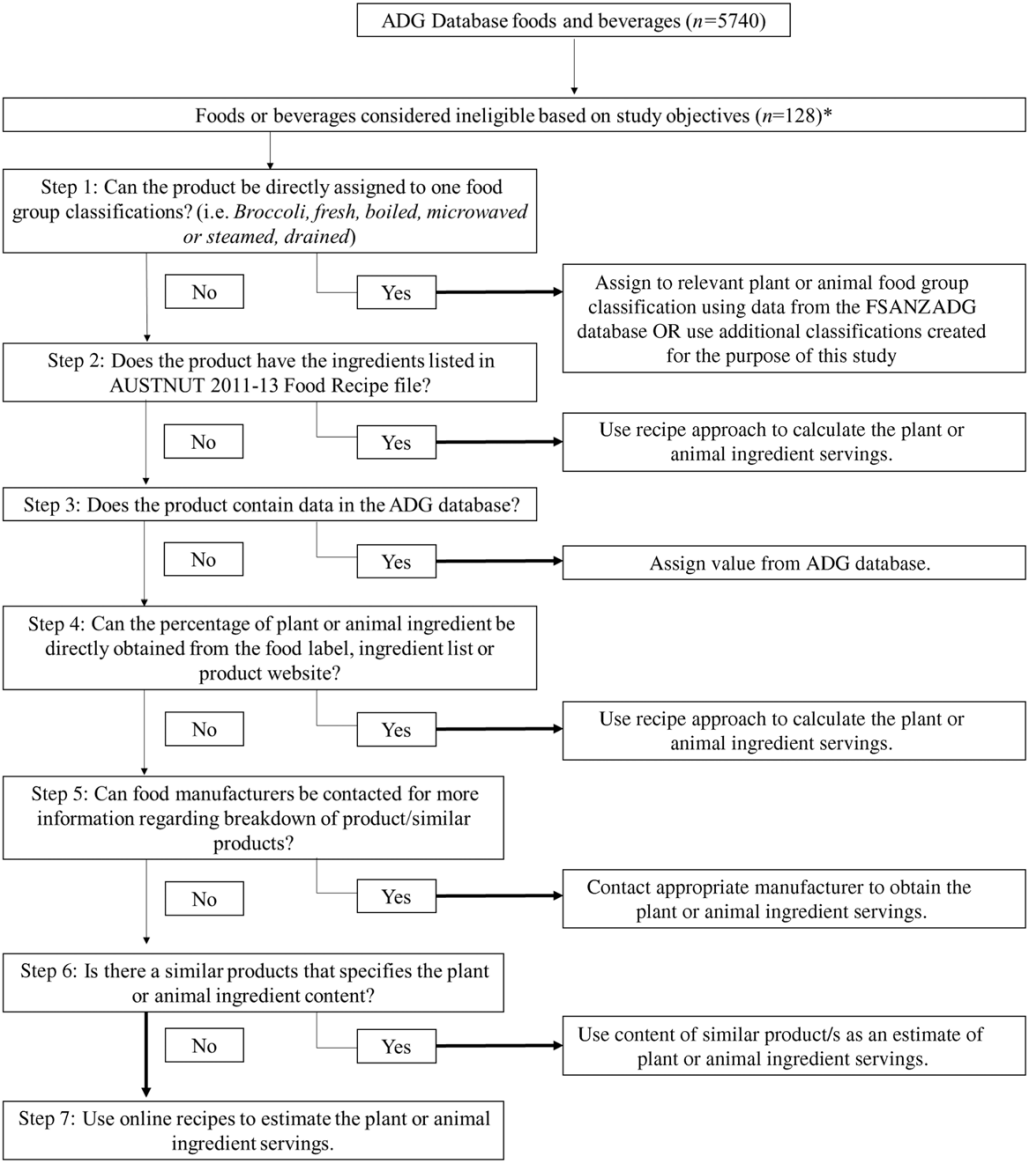
The systematic approach to determining plant and animal content of foods and beverages within the ADG database. *Products that could not be directly classified into any of the twenty-three plant and animal food group classifications, including water and alcoholic beverages (*n* 52). ADG = Australian Dietary Guidelines.

The first phase in the database development was defining the broader plant and animal food group classifications to capture most of the AUSNUT 2011–2013 food and beverage items. Published plant-based diet quality indexes^(5,6)^ were first consulted to determine whether they could be matched with existing food group classifications already used in the ADG database, for example, whole grains, refined grains, fruits and vegetables. Each of the 5740 food and beverage items was then reviewed. Food and beverage items that were not classified in the ADG database were examined to confirm if one or more of the ingredients could be coded within an existing ADG food group classification or whether the creation of a new category was required. For example, a new ‘*tea and coffee’* category was created to group unclassified items such as *Tea, herbal, other, without milk* and *Coffee, espresso-style, without milk.* Similarly, ‘*animal fats’,* another food group classification, was created to categorise unclassified products such as *Butter, plain, salted* and *Ghee, clarified butter.* Throughout this initial review process, certain AUSNUT 2011–2013 items were considered irrelevant to the objectives of this study, as they did not directly fit into an existing food category and were believed not to warrant the creation of a new food group classification. Therefore, certain items remained unclassified (*n* 128), including non-nutritive sweeteners, human breast milk and infant formulas, protein powders, nutritional supplements, yeasts, and cooking additives such as salt, pectin, or gelatine (Fig. 1). While not considered as a plant or animal ingredient, the water and alcohol content of all foods and beverages, including multi-ingredient products and mixed dishes, were still separately classified in the database. Following consensus amongst the research team, a total of twenty-three plant and animal food group classifications were defined (Table 1).

**Table 1.** Finalised plant and animal food groups used for the database development

Plant-based food groups	Serving Size‡	Example of plant or plant-derived ingredients	Animal-based food groups	Serving Size‡	Example of animal or animal-derived ingredients
Whole grains† (101)	30–120 g	Brown rice, barley, buckwheat, sorghum, quinoa, whole oats, wholegrain or wholemeal wheat flour-based (bread, pasta)	Animal fats*	600 kJ Equiv.	Butter, lard, ghee, shortening animal fat, suet added in cooking, cream, sour cream.
Fruits† (301–2)	30–150 g	Apple, banana, pear, orange, strawberry, grapes, other whole, sliced or diced, fresh, frozen, canned or dried fruit	Low-fat dairy† (403)	26–260 g	Reduced-fat or skim milk, buttermilk, reduced-fat ricotta and cottage, quark, most regular and reduced-fat natural or Greek-style yogurt.
Vegetables†, herbs and spices* (201–3205)	5–130 g	Broccoli, cauliflower, kale, peas, beans, asparagus, snow peas, lettuce, silverbeet, potato, sweet potato, fresh and dried herbs and spices, 100 % vegetable juice	Moderate-fat dairy† (402)	40–260 g	Regular fat/full cream milk, reduced-fat processed cheddar
Nuts and seeds† (508)	30 g	Nuts, seeds, nut butters, tahini, nut/seed pastes, nut portion of nut milks	High-fat dairy† (401)	26–40 g	Parmesan, camembert, brie, cheddar, regular fat cream cheese, halloumi
Legumes† (204)	75 g	Cooked dried or canned beans, baked beans, chickpeas, split peas, lentils	Eggs† (506)	120 g	Whole eggs, egg yolks and egg whites
Unsaturated plant oils and spreads† (701–2)	7–10 g	Olive oil and other polyunsaturated and monounsaturated oils and margarine spreads	Fish and seafood† (505)	100 g	Fresh and canned fish, prawns, lobster, mussels, oysters, scallops and clams
Tea and coffee*	50 ml espresso or 2 g instant coffee or dry tea powders, 250 ml brewed tea (no milk) Equiv.	Black tea, green tea, herbal tea, coffee	Red meats, lean† (501)	65 g	Cooked lean red meats with a fat content <10 % such as beef, lamb, veal, pork, goat, rabbit, venison, kangaroo and lean (lower salt) sausages
Refined grains† (102)	30–120 g	White wheat flour-based bread or pasta, flatbread, rolls, cooked white rice, polenta, couscous, sago, cornflakes	Red meats, non-lean† (502)	65 g	Cooked red meats with a fat content ≥ 10 % such as beef, lamb, veal, pork, goat, rabbit, venison, kangaroo and lean (lower salt) sausages
Fruit juices† (303)	130 g	100 % Fruit juices	Poultry, lean† (503)	80 g	Cooked chicken, turkey, goose, duck and quail with fat content < 10 %
Saturated plant fats*	600 kJ Equiv.	Palm oil, coconut oil, coconut cream, coconut milk, shortening vegetable fats	Poultry, non-lean† (504)	80 g	Chicken, turkey, goose, duck, quail with fat content ≥ 10 % fat or processed luncheon meat
Sugars and syrups§	Added sugars (1 g)	Sugar (white, brown, raw), agave syrup, maple syrup	Miscellaneous animal products*	1 g (honey only), 120 mg Na or 600 kJ Equiv.	Stocks (chicken, beef, fish), bonox (beef extract), Fish sauce, oyster sauce, honey.
Miscellaneous plant products*	120 mg Na or 600 kJ Equiv.	Soy sauce, vegetable stock, yeast and vegetable spreads (Vegemite/Promite)			

* New food classification created for the purpose of this study.

† The number in brackets refers to the existing food classifications present in the ADG database^(7)^.

‡ Serving sizes varied within food group classification depending on the product. Please refer to supplementary materials for a further breakdown of specific serving sizes within each food group.

§ Applied existing ‘added sugars’ category available in the AUSNUT 2011–2013 Food Nutrient Database file^(10)^.

Details relating to the food servings for each of the 23 food group classifications are provided in Tables 1 and in the first excel sheet of the database (link available in Item 1 – online Supplementary Materials). Applicable food serving size classifications that already existed within the ADG database remained unchanged (Table 1). The ADG uses a range of serving sizes to classify different types of foods within each food group (i.e. bread *v*. pasta) to reflect similar amounts of kilojoules and/or key nutrients, as well as the amounts of food commonly consumed in Australia, which facilitates translation and accuracy^(13)^. To maintain consistency with this approach when expanding the ADG database^(7)^, unclassified food items and ingredients were similarly expressed in terms of the serving size for each plant or animal food group per 100 grams. In developing the new food group classifications, serving sizes were based on nutritional guidelines or recommendations relevant to unclassified food and beverage categories. For instance, a standard serving for animal and saturated plant fats equated to 600 kJ, reflecting the ADG recommendations for the discretionary foods category where these products fit^(9)^. This was the same for some foods within the defined miscellaneous plant and animal food groups. However, for particular foods within these categories, such as stocks and sauces that are not necessarily high in energy (kJ) but are high in undesirable nutrients such as Na, the target of 120 mg Na/100 g aligns with FSANZ guidelines for low salt products^(14)^ was deemed more relevant. Coffee and tea were standardised to calculate the relevant non-milk proportions. Added sugars were selected over free sugars, as free sugars include sugars naturally present in products like honey, fruit juices and fruit juice concentrates^(15)^. This would have resulted in duplication with other food groups and not allowed for differentiation between plant and animal-derived ingredients (i.e., honey). Further details of the specific serving sizes for sub-groups within each plant and animal food group are summarised in item 1 of the Supplementary materials.

### Identifying and calculating plant and animal content of foods and beverages

The methods for identifying and calculating plant and animal content of foods and beverages within this database were guided by the methods used previously in the development of a nut and cereal fibre database^(16,17)^. This database was created using a systematic approach outlined below and illustrated in Fig. 1. The systematic approach was undertaken for each food group classification outlined in Table 1, except for added sugars, as this had already been undertaken for each NNPAS product^(18)^.

After defining the main plant and animal food group classifications, the proportion of plant and animal content in each product was then determined. The AUSNUT 2011–2013 foods and beverages were first reviewed to identify whether they contained any plant or animal ingredients for each food group classification outlined in Table 1. Foods that could only be classified directly into one food group, for instance, *Barley, pearl, uncooked*, was assigned to the whole grains food group and received a value of 0 for all other food groups. If uncertain whether the product could fit within only one of the food group classifications, information available in the AUSNUT 2011–2013 Food Details database file^(11)^, AUSNUT 2011–2013 Food Recipe File^(12)^, or based on the product name (i.e. *Pastry, puff, with butter, commercial, raw)* was consulted to make an informed decision. The weight change percentage factors available in the AUSNUT Food Recipe^(12)^ were used to account for weight changes during food processing and cooking (i.e. moisture loss). This was also relevant for some foods and food groups, where the serving sizes used per the ADG (Table 1) refers to the cooked or prepared portion of a product before consumption. Therefore, the weight change percentage factors helped to convert the raw or uncooked ingredients, for instance, the uncooked, dried or raw grains, legumes, vegetables, meat, poultry and seafood, to the equivalent number of serving size as the cooked version^(7)^.

To calculate the plant or animal content of multi-ingredient foods required a recipe-based approach using the AUSNUT 2011–2013 Food Recipe File^(12)^ for individual ingredient composition (Table 2). For many multi-ingredient products and mixed dishes, the plant- or animal-containing ingredients were identified initially and then calculated to determine the final content of the product (Table 2).

**Table 2.** Calculating plant or animal food group content using the recipe-based approach

Product name	Weight factor	Ingredients	Applicable food group for ingoing ingredient	Food group serving/100 g of ingoing ingredient	Ingredient weight (g)	Proportion of ingredient†	Food group serving/100 g of outgoing ingredient‡ or multi-ingredient product§	Total food group(s) serving/100 g for product||
Calculation of the plant and/or animal content in a multi-ingredient food (e.g. hollandaise sauce)								
Sauce, hollandaise, homemade	–10	Butter, plain, no added salt	Animal fats	5·045	125	0·67 206	3·39	Animal fats: 3·39
		Pepper, ground, black or white	Vegetables, herbs and spices	20	0·34	0·00183	0·037	Vegetables, herbs and spices: 0·037
		Egg, chicken, yolk, raw	Eggs	0·731	51	0·2742	0·200	Eggs: 0·200
		Juice, lemon, commercial	Fruit juices	0·769	10·32	0·05549	0·043	Fruit juices: 0·043
		Water, tap	Water	0·4	20	0·10 753	0·043	0·043
					185·994*			
Calculation of plant and animal content in a mixed dish (e.g. eggs benedict) when the ingredients contain another multi-ingredient food (e.g. hollandaise sauce)								
Eggs Benedict, poached chicken egg, with smoked salmon, spinach and hollandaise sauce, with bread or English muffin	0	Sauce, hollandaise, homemade			25·5	0·19 570		Animal fats: 0·756
		Butter, plain, no added salt	Animal fats	3·39			0·664	Vegetables, herbs and spices: 0·300
		Pepper, ground, black or white	Vegetables, herbs and spices	0·037			0·007	Eggs: 0·234
		Egg, chicken, yolk, raw	Eggs	0·200			0·039	Fruit juices: 0·008
		Juice, lemon, commercial	Fruit juices	0·043			0·008	Fish and seafood: 0·083
		Water, tap	Water	0·043			0·008	Refined grains: 0·798
		Egg, chicken, whole, poached, no added fat	Eggs	0·833	30·5	0·23 408	0·195	
		Butter, not further defined	Animal fats	5·045	2·4	0·01842	0·091	
		Salmon, smoked, sliced	Fish and seafood	1·000	10·8	0·08289	0·083	
		Spinach, boiled, microwaved or steamed, drained, not further defined	Vegetables, herbs and spices	1·333	27·6	0·21 182	0·282	
		Muffin, English style, from white flour, commercial, toasted	Refined grains	3·106	33·5	0·25 710	0·798	
					103·3*			

* Final weight of the product = sum of all ingredients (g) × .

†Proportion of ingredient 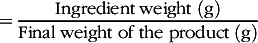 . Ingredient proportions are calculated after accounting for changes in weight (weight factor) during food processing and cooking of the final product.

‡Food group servings/100 g of outgoing ingredient = portion of ingredient × food group serving/100 g of ingoing ingredient.

§Food group servings/100 g of outgoing multi-ingredient product 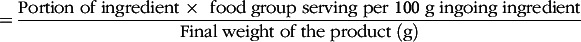

||Total food group servings/100 g for the product = sum of all food servings for each unique food group in the product.

For those animal or plant-containing foods in the AUSNUT 2011–2013 Food Details database file^(11)^ that did not have ingredients listed in the AUSNUT 2011–2013 Food Recipe file^(12)^ (such as *Breakfast cereal, mixed grain (wheat, corn, rice & oat), flakes & clusters, honey & macadamias, added vitamins B*_*1*_ *, B*_*2*_ *, B*_*3*_ *, E & folate, Ca & Fe*), alternative methods were used. The AUSNUT 2011–2013 Food Details database file^(11)^ was used to determine the specific commercial products used as a reference for that product. For example, the food *Breakfast cereal, mixed grain (wheat, corn, rice & oat), flakes & clusters, honey & macadamias, added vitamins B*_*1*_ *, B*_*2*_ *, B*_*3*_ *, E & folate, Ca & Fe* had Sanitarium Lite ‘n’ Tasty Macadamia and Honey with Oat Clusters listed as the reference commercial product. When available, the unclassified plant or animal content was calculated based on the product label found on the food packaging of these specific commercial products obtained from local grocery stores or via manufacturers’ websites. Similar products were used where the specifically referenced commercial products were unavailable. A label-based method was used to determine the unclassified plant or animal content in these instances when commercial products included the exact proportion of the plant or animal ingredient on the label (Table 3). If available label data could not be used to make an accurate estimation, manufacturers were contacted for a detailed breakdown of ingredients.

**Table 3.** Calculating plant or animal food group content using the label-based approach

Product name	Matching commercial product*	Ingredients as per label	Ingredient amount as per label (%)	Input status†	Applicable food group for ingoing ingredient	Food group serving/100 g of ingoing ingredient‡	Unique food group(s) applicable to product§
Breakfast cereal, mixed grain (wheat, corn, rice and oat), flakes and clusters, honey and macadamias, added vitamins B_1_, B_2_, B_3_, E and folate, Ca and Fe	Sanitarium Lite ‘n’ tasty Macadamia and Honey with Oat Clusters||	Cereals [wheat, oats (10 %), whole wheat flour), corn, puffed wheat, wheat bran, rice]	69 %	Classified	–	–	–
		Sugar	6–10 %	Classified	–	–	–
		Macadamia nuts	6 %	Classified	–	–	–
		Maltodextrin	2–6 %	N/A	–	–	–
		Honey	2 %	Unclassified	Miscellaneous animal products	100	2
		Salt¶	0·74 %	N/A	–	–	–
		Barley malt extract	<0·74 %	N/A	–	–	–
		Vitamins	<0·74 %	N/A	–	–	–
		Minerals	<0·74 %	N/A	–	–	–

* Located using Food Standards Australia New Zealand AUSNUT 2011–2013 Food Details file^(11)^.

† Classified = Ingredient has already assigned a classification as per the selected food group serves from the ADG database; Unclassified = Ingredient that has not yet been classified. Only unclassified ingredients were updated when using the label-based approach.

‡ Using recipe approach for matching ingredients within ADG Database^(7)^.

§ Already assigned food group servings for classified ingredients were used. For unclassified ingredients, total food group(s) serving/100 g for product = ingredient amount (%) × food group serving/100 g of ingoing ingredient. The sum of food serving/100 g for each unique food group.

|| Sanitarium Health Food Co., Berkeley Vale, NSW.

¶ Based on 295 mg Na/100 g product (food label data).

When none of the above methods was feasible, the plant and/or animal content was determined either using a comparable product already available within the ADG database^(7)^ or standard recipes available online from Australian websites (for example, *taste.com.au* and *delicious.com.au*), which were selected based on professional judgement. The food group servings for any unclassified ingredients were calculated manually, and the proportion was applied to the database using the recipe-based approach.

A 20 % random sample of ADG foods was identified to mitigate the risk of error, and a second researcher independently cross-checked the methods applied above.

### Data analysis

Following the development of the database, the number of plant- and animal-containing products was calculated and presented according to AUSNUT 2011–2013 major food groups^(19)^. Additionally, the discretionary food list^(20)^ generated by the ABS was used to explore the number of plant- and animal-containing products across core and discretionary foods.

Finally, the original ADG^(7)^ and the updated expanded database were applied to a random twenty participants of the NNPAS^(8)^ to illustrate the difference in distribution between the twenty-three food groups from core and discretionary products. A smaller sample size was intentionally selected to illustrate the inter-individual variation between the databases. To manage data and generate figures, R version 3.5.0 was used.

## Results

Out of the 5612 AUSNUT 2011–2013 foods and beverages classified in the ADG database, 4687 (83·5 %) were determined to be plant-based or plant-containing products. Of these, 68·2 % were entirely derived from plants. In contrast, 3701 (65·9 %) of the foods and beverages were classified as animal-based or animal-containing products, with 34·05 % being entirely animal-based. The plant or animal content of over 90 % of the foods and beverages was calculated using the recipe-based approach. The content of remaining foods or beverages was estimated using either the label-based method, based on a similar product or online recipes. A link to the final database is provided in the manuscript’s Supplementary materials (Item 1).

The proportions of plant and animal content varied in products across AUSNUT 2011–2013 major food groups. However, the most notable observation was how plant- and animal-derived ingredients were so widely dispersed across a range of these major food groups (Table 4). For example, animal fats, which were previously not classified, were present in 34·2 % of products within the ‘fats and oils’ category and 53·2 % of products of the ‘cereal-based products and dishes’. Interestingly, plant and animal-containing products were also distributed across both discretionary and core foods. For instance, while fruits and nuts and seeds are considered core foods themselves, they were also identified as ingredients in several less healthy, discretionary products (Table 5).

**Table 4.** The number and percentage of food and beverage items (*n* 5740) within each of the AUSNUT 2011–2013 two-digit major food groups that were found to contain plant and/or animal ingredients*

	Plant food groups (*n*, (%))																							
	Whole grains		Fruits		Vegetables, herbs and spices		Nuts and seeds		Legumes		Unsaturated plant oils and spreads		Tea and coffee		Refined grains		Fruit juices		Saturated plant fats		Sugars and syrups		Miscellaneous plant products	
AUSNUT 2011–2013 major food name	*n*	%	*n*	%	*n*	%	*n*	%	*n*	%	*n*	%	*n*	%	*n*	%	*n*	%	*n*	%	*n*	%	*n*	%
Non-alcoholic beverages	6	1·9	7	2·2	20	6·2	2	0·6	13	4	28	8·7	94	29·2	16	5	128	39·8	0	0	196	60·9	0	0
Cereals and cereal products	283	56·7	80	16	24	4·8	96	19·2	5	1	99	19·8	0	0	275	55·1	3	0·6	18	3·6	181	36·3	3	0·6
Cereal-based products and dishes	180	19·7	240	26·3	503	55	99	10·8	46	5	648	70·9	7	0·8	890	97·4	72	7·9	252	27·6	740	81	158	17·3
Fats and oils	0	0	0	0	1	0·9	0	0	0	0	94	82·5	0	0	0	0	0	0	11	9·6	0	0	0	0
Fish and seafood products and dishes	9	2·9	4	1·3	59	19·2	2	0·7	0	0	180	58·6	0	0	93	30·3	5	1·6	117	38·1	51	16·6	13	4·2
Fruit products and dishes	4	1·5	245	91·1	4	1·5	9	3·3	0	0	12	4·5	0	0	5	1·9	25	9·3	3	1·1	108	40·1	0	0
Egg products and dishes	0	0	1	1·1	55	60·4	0	0	19	20·9	37	40·7	0	0	5	5·5	5	5·5	0	0	17	18·7	0	0
Meat, poultry and game products and dishes	42	3·3	28	2·2	234	18·6	28	2·2	22	1·8	555	44·2	0	0	241	19·2	8	0·6	363	28·9	186	14·8	92	7·3
Milk products and dishes	4	1·2	70	20·2	7	2	13	3·8	1	0·3	16	4·6	12	3·5	49	14·2	9	2·6	13	3·8	210	60·7	0	0
Dairy and meat substitutes	6	11·3	4	7·5	4	7·5	2	3·8	48	90·6	26	49·1	1	1·9	7	13·2	0	0	4	7·5	39	73·6	3	5·7
Soup	15	16·1	0	0	72	77·4	0	0	20	21·5	56	60·2	0	0	64	68·8	1	1·1	41	44·1	64	68·8	48	51·6
Seed and nut products and dishes	3	3·9	4	5·2	3	3·9	63	81·8	1	1·3	29	37·7	0	0	0	0	3	3·9	6	7·8	10	13	1	1·3
Savoury sauces and condiments	3	1·9	17	10·8	128	81	14	8·9	7	4·4	84	53·2	0	0	54	34·2	39	24·7	6	3·8	113	71·5	51	32·3
Vegetable products and dishes	9	1·4	3	0·5	654	99·8	10	1·5	6	0·9	277	42·3	0	0	47	7·2	10	1·5	116	17·7	57	8·7	14	2·1
Legume and pulse products and dishes	8	14·8	0	0	16	29·6	1	1·9	52	96·3	14	25·9	0	0	8	14·8	1	1·9	7	13	20	37	2	3·7
Snack foods	14	26·4	0	0	28	52·8	4	7·5	2	3·8	42	79·2	0	0	37	69·8	0	0	0	0	34	64·2	1	1·9
Sugar products and dishes	0	0	34	40	6	7·1	1	1·2	0	0	9	10·6	2	2·4	6	7·1	24	28·2	9	10·6	81	95·3	4	4·7
Confectionery and cereal/nut/fruit/ seed bars	34	23·6	42	29·2	2	1·4	49	34	0	0	61	42·4	2	1·4	42	29·2	7	4·9	8	5·6	139	96·5	0	0
Alcoholic beverages	0	0	0	0	6	8·7	0	0	0	0	6	8·7	5	7·2	7	10·1	11	15·9	6	8·7	21	30·4	0	0
Special dietary foods	16	19·3	0	0	1	1·2	0	0	17	20·5	31	37·3	0	0	31	37·3	2	2·4	0	0	56	67·5	0	0
Miscellaneous	0	0	0	0	22	28·6	0	0	0	0	0	0	0	0	2	2·6	0	0	0	0	18	23·4	14	18·2
Infant formulae and foods	1	7·1	2	14·3	1	7·1	0	0	0	0	1	7·1	0	0	5	35·7	2	14·3	1	7·1	2	14·3	0	0
Reptiles, amphibia and insects	0	0	0	0	0	0	0	0	0	0	0	0	0	0	0	0	0	0	0	0	0	0	0	0

	Animal food groups (*n*, (%))																					
	Animal fats		Low-fat dairy		Moderate-fat dairy		High-fat dairy		Eggs		Fish and seafood		Red meats, lean		Red meats, non-lean		Poultry, lean		Poultry, non-lean		Miscellaneous non-plant products	
AUSNUT 2011–2013 major food name	*n*	%	*n*	%	*n*	%	*n*	%	*n*	%	*n*	%	*n*	%	*n*	%	*n*	%	*n*	%	*n*	%
Non-alcoholic beverages	1	0·3	36	11·2	30	9·3	25	7·8	1	0·3	0	0	0	0	0	0	0	0	0	0	0	0
Cereals and cereal products	20	4	6	1·2	38	7·6	15	3	26	5·2	0	0	1	0·2	3	0·6	0	0	0	0	33	6·6
Cereal-based products and dishes	486	53·2	99	10·8	254	27·8	358	39·2	503	55	111	12·1	179	19·6	140	15·3	94	10·3	5	0·5	129	14·1
Fats and oils	39	34·2	0	0	0	0	0	0	0	0	0	0	0	0	0	0	0	0	0	0	0	0
Fish and seafood products and dishes	123	40·1	3	1	30	9·8	6	2	40	13	307	100	0	0	0	0	0	0	0	0	15	4·9
Fruit products and dishes	7	2·6	2	0·7	1	0·4	2	0·7	1	0·4	0	0	0	0	0	0	0	0	0	0	0	0
Egg products and dishes	42	46·2	2	2·2	44	48·4	21	23·1	91	100	7	7·7	8	8·8	11	12·1	3	3·3	0	0	2	2·2
Meat, poultry and game products and dishes	458	36·5	8	0·6	48	3·8	5	0·4	137	10·9	6	0·5	717	57·1	261	20·8	215	17·1	48	3·8	93	7·4
Milk products and dishes	69	19·9	186	53·8	132	38·2	134	38·7	51	14·7	0	0	0	0	0	0	0	0	0	0	18	5·2
Dairy and meat substitutes	5	9·4	2	3·8	1	1·9	1	1·9	1	1·9	1	1·9	0	0	0	0	0	0	0	0	2	3·8
Soup	44	47·3	9	9·7	7	7·5	2	2·2	16	17·2	9	9·7	18	19·4	11	11·8	17	18·3	1	1·1	51	54·8
Seed and nut products and dishes	0	0	0	0	1	1·3	0	0	0	0	0	0	0	0	0	0	0	0	0	0	3	3·9
Savoury sauces and condiments	27	17·1	13	8·2	16	10·1	27	17·1	29	18·4	16	10·1	0	0	2	1·3	0	0	0	0	41	25·9
Vegetable products and dishes	174	26·6	1	0·2	15	2·3	40	6·1	26	4	6	0·9	12	1·8	17	2·6	10	1·5	0	0	24	3·7
Legume and pulse products and dishes	7	13	0	0	0	0	2	3·7	3	5·6	0	0	0	0	2	3·7	0	0	0	0	0	0
Snack foods	4	7·5	1	1·9	1	1·9	17	32·1	1	1·9	1	1·9	0	0	0	0	0	0	0	0	0	0
Sugar products and dishes	16	18·8	8	9·4	3	3·5	5	5·9	8	9·4	0	0	0	0	0	0	0	0	0	0	1	1·2
Confectionery and cereal/nut/fruit/ seed bars	11	7·6	43	29·9	17	11·8	60	41·7	10	6·9	0	0	0	0	0	0	0	0	0	0	4	2·8
Alcoholic beverages	7	10·1	6	8·7	6	8·7	0	0	1	1·4	0	0	0	0	0	0	0	0	0	0	0	0
Special dietary foods	0	0	49	59	23	27·7	10	12	0	0	0	0	0	0	0	0	0	0	0	0	0	0
Miscellaneous	1	1·3	0	0	1	1·3	1	1·3	0	0	0	0	0	0	0	0	0	0	0	0	8	10·4
Infant formulae and foods	1	7·1	1	7·1	4	28·6	0	0	1	7·1	0	0	1	7·1	0	0	1	7·1	0	0	0	0
Reptiles, amphibia and insects	0	0	0	0	0	0	0	0	0	0	0	0	6	85·7	0	0	0	0	0	0	1	14·3

* Plant and animal ingredients are summarised into twenty-three plant and animal food groups (shown in Table 1).

**Table 5.** The number and percentage of core and discretionary foods and beverages that were found to contain plant and/or animal ingredients*

	Core foods and beverages		Discretionary foods and beverages	
	*n*	%	*n*	%
Whole grains	533	13	104	6·4
Fruits	496	12·1	285	17·4
Vegetables, herbs and spices	1420	34·6	430	26·3
Nuts and seeds	250	6·1	143	8·7
Legumes	225	5·5	34	2·1
Unsaturated plant oils and spreads	1541	37·5	764	46·7
Tea and coffee	90	2·2	33	2
Refined grains	1048	25·5	836	51·1
Fruit juices	132	3·2	223	13·6
Saturated plant fats	784	19·1	197	12
Sugars and syrups	1147	27·9	1196	73·1
Miscellaneous plant products	312	7·6	92	5·6
Animal fats	1006	24·5	536	32·8
Low-fat dairy	231	5·6	244	14·9
Moderate-fat dairy	366	8·9	306	18·7
High-fat dairy	378	9·2	353	21·6
Eggs	503	12·3	443	27·1
Fish and seafood	357	8·7	107	6·5
Red meats, lean	859	20·9	83	5·1
Red meats, non-lean	332	8·1	115	7
Poultry, lean	311	7·6	29	1·8
Poultry, non-lean	44	1·1	10	0·6
Miscellaneous animal-based products	351	8·6	74	4·5
Total number of AUSNUT 2011–2013 items (*n*)	4104		1636	

* Plant and animal ingredients are summarised into twenty-three plant and animal food groups (shown in Table 1).

**Fig. 2.**
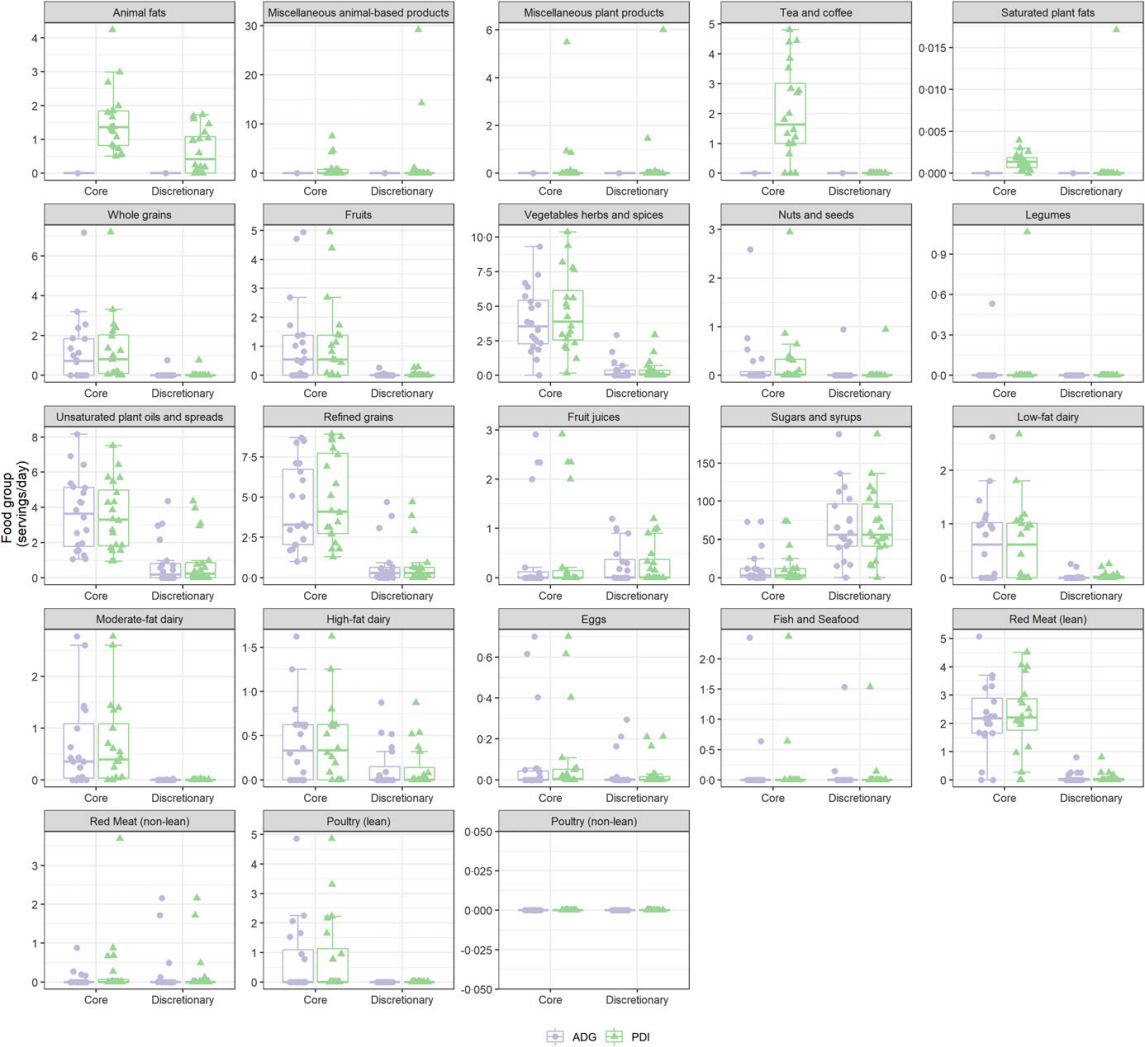
The distribution of the twenty-three food groups from core and discretionary products when the ADG and updated database are applied to data from *n* 20 participants from the NNPAS. ADG = Australian Dietary Guidelines; NNPAS = National Nutrition and Physical Activity Survey.

There were differences in distribution between the twenty-three food groups from core and discretionary products when applying the different databases to the same participants (Fig. 2). The new database captures commonly consumed foods such as animal fats, saturated plant fats, tea and coffee that are unclassified in the original ADG database. Furthermore, intakes of some food groups, such as vegetables, herbs, spices, nuts and seeds, as well as refined grains, were higher in the updated database. This is likely due to the original ADG database overlooking the content in some multi-ingredient products and mixed dishes.

## Discussion

This study outlines the systematic approach utilised to expand an existing database to provide quantitative estimates of the plant and animal content of Australian foods, beverages and dishes. To our knowledge, this type of database is the first of its kind. Prior to the development of this database, there was no way to accurately assess the amount of plant and animal intakes without manually quantifying each food and beverage item individually. The database addresses this gap and alleviates resource burden by providing a comprehensive and readily accessible option for accurately evaluating both the intake and quality of plant- and animal-based foods.

To best assess diet quality, it is pertinent to consider the context in which certain foods are eaten. For example, people consume vegetables in various ways, including whole vegetables, vegetable juices, salads, sandwich fillings, stir fries, pizzas and pies. This study demonstrated the versatility of plant and animal ingredients as they were found in various foods across many food categories, including savoury and sweet foods as well as discretionary and core foods. Surprisingly, fruits, nuts and seeds were present in a greater percentage of discretionary products than in core foods and beverages. Moreover, over 97 % of animal fat-containing foods were found in major food groups outside of the AUSNUT 2011–2013 ‘fats and oils’ group, highlighting the need for a database that considers animal and plant-based ingredients found in multi-ingredient food and mixed dishes.

Accurate estimation of plant and animal food consumption is particularly important when teasing out the associated benefits of plant-based diets. Plant-based diets have been associated with many health benefits, including reduced cardiovascular disease risk, improved weight maintenance, blood pressure and cholesterol levels^(21–24)^. However, much of this evidence has been obtained from participants who self-report that they follow a vegan or vegetarian eating pattern or from studies that use FFQ that focus on whole food consumption, with limited exploration of plant or animal intakes from multi-ingredient foods and mixed dishes. The present study demonstrated that the new database can capture intakes of some plant and animal foods that were either unclassified or overlooked in the original ADG database. Investigations into the total plant and animal intakes within the entire diet (from whole foods, multi-ingredient foods and mixed dishes) would produce a more accurate estimation of the dose or ratio of plant to animal foods associated with more favourable health outcomes. Further, this database can highlight where individuals consume most of their plant and animal foods in their diet and can therefore inform more targeted dietary advice and interventions to improve overall diet quality.

The development of this database was strengthened by using a systematic approach to ensure the accuracy of the data presented. This systematic approach ensured the same methods were applied to each food or beverage item and that approximations were limited by the stringent process. However, the development of this database is not without limitations. There is potential for human error to incorrectly classify foods or calculate the plant or animal content given that the database contains 5740 foods and beverages. However, a second researcher independently cross-checked a random 20 % sample to reduce the risk of error. Recipes described in AUSNUT 2011–2013 Recipe file^(12)^ may differ from recipes used by individuals, thereby potentially altering the content of some multi-ingredient foods and dishes. Furthermore, the label information of products used may differ amongst brands and batches of relevant commercial products. Assumptions were also required when exercising professional judgement to select similar products or recipes when estimating the plant and animal content of certain foods and beverages. Another limitation is that the difference observed between the databases is dependent on the variation of participants’ intakes. For example, no distinction may be noticeable if participants do not eat a varied diet, including those food and beverage items that were initially unclassified or partly classified in the original ADG database. Finally, this database may require modifications given changes in the food supply over time and differences amongst countries. Nevertheless, this paper provides a systematic process suitable for applying future updates or developing other novel food databases.

### Conclusion

This database is the first of its kind in Australia and was developed to allow quantitative estimates of plant- and animal-based intakes amongst the Australian population. The database can also assess plant-based diet quality and relevant health outcomes which will be of use in future epidemiological and experimental research.

## Supplementary material

For supplementary material accompanying this paper visit https://doi.org/10.1017/S0007114523001101. click here to view supplementary material
