# Supplementary material for: Expansion of an Australian food composition database to estimate plant and animal intakes
Source: Br J Nutr. 2023 May 9;130(11):1950–60. doi: 10.1017/S0007114523001101 (PMC10630146; doi:10.1017/S0007114523001101)
Supplement: Supplementary file 1 [file S0007114523001101sup001.pdf]

Supplementary materials:
Expansion of an Australian food composition database to estimate plant and animal intakes

Item 1. Link to access and download the Australian Plant-Based Food Composition Database:

https://osf.io/f6j7e/?view_only=5767089e44834071b32b1e152541ef1b
